# Epigenome‐wide three‐way interaction study identifies a complex pattern between *TRIM27*, *KIAA0226*, and smoking associated with overall survival of early‐stage NSCLC

**DOI:** 10.1002/1878-0261.13167

**Published:** 2022-01-07

**Authors:** Xinyu Ji, Lijuan Lin, Juanjuan Fan, Yi Li, Yongyue Wei, Sipeng Shen, Li Su, Andrea Shafer, Maria Moksnes Bjaanæs, Anna Karlsson, Maria Planck, Johan Staaf, Åslaug Helland, Manel Esteller, Ruyang Zhang, Feng Chen, David C. Christiani

**Affiliations:** ^1^ Department of Biostatistics Center for Global Health School of Public Health Nanjing Medical University Nanjing China; ^2^ Department of Biostatistics University of Michigan Ann Arbor MI USA; ^3^ Department of Environmental Health Harvard T.H. Chan School of Public Health Boston MA USA; ^4^ China International Cooperation Center for Environment and Human Health Nanjing Medical University Nanjing China; ^5^ Pulmonary and Critical Care Division Department of Medicine Massachusetts General Hospital and Harvard Medical School Boston MA USA; ^6^ Department of Cancer Genetics Institute for Cancer Research Oslo University Hospital Oslo Norway; ^7^ Division of Oncology Department of Clinical Sciences Lund and CREATE Health Strategic Center for Translational Cancer Research Lund University Lund Sweden; ^8^ Institute of Clinical Medicine University of Oslo Oslo Norway; ^9^ Josep Carreras Leukaemia Research Institute Barcelona Spain; ^10^ Centro de Investigacion Biomedica en Red Cancer Madrid Spain; ^11^ Institucio Catalana de Recerca i Estudis Avançats Barcelona Spain; ^12^ Physiological Sciences Department School of Medicine and Health Sciences University of Barcelona Barcelona Spain; ^13^ State Key Laboratory of Reproductive Medicine Nanjing Medical University Nanjing China; ^14^ Jiangsu Key Lab of Cancer Biomarkers, Prevention and Treatment Cancer Center Collaborative Innovation Center for Cancer Personalized Medicine Nanjing Medical University Nanjing China

**Keywords:** DNA methylation, non‐small‐cell lung cancer, overall survival, prognostic prediction, three‐way interaction

## Abstract

The interaction between DNA methylation of tripartite motif containing 27 (cg05293407*_TRIM27_*) and smoking has previously been identified to reveal histologically heterogeneous effects of *TRIM27* DNA methylation on early‐stage non‐small‐cell lung cancer (NSCLC) survival. However, to understand the complex mechanisms underlying NSCLC progression, we searched three‐way interactions. A two‐phase study was adopted to identify three‐way interactions in the form of pack‐year of smoking (number of cigarettes smoked per day × number of years smoked) × cg05293407*_TRIM27_* × epigenome‐wide DNA methylation CpG probe. Two CpG probes were identified with FDR‐*q* ≤ 0.05 in the discovery phase and *P* ≤ 0.05 in the validation phase: cg00060500*_KIAA0226_* and cg17479956*_EXT2_*. Compared to a prediction model with only clinical information, the model added 42 significant three‐way interactions using a looser criterion (discovery: FDR‐*q* ≤ 0.10, validation: *P* ≤ 0.05) had substantially improved the area under the receiver operating characteristic curve (AUC) of the prognostic prediction model for both 3‐year and 5‐year survival. Our research identified the complex interaction effects among multiple environment and epigenetic factors, and provided therapeutic target for NSCLC patients.

## Graphical abstract

We performed a two‐phase designed epigenome‐wide three‐way gene‐smoking interaction study of NSCLC survival and identified two CpG probes (cg00060500*_KIAA0226_* and cg17479956*_EXT2_*), together with pack‐year of smoking and cg05293407*_TRIM27_*, exhibiting significant three‐way interaction effects. Meanwhile, these significant three‐way interactions substantially improved the area under the receiver operating characteristic curve (AUC) of the prognostic prediction model for both 3‐ and 5‐year survival.

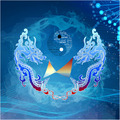

**Abbreviations**
AUC: area under the receiver operating characteristic curve
CI: confidence interval
C‐index: concordance index
DEGs: differentially expressed genes
FDR: false discovery rate
GO: gene ontology
HR: hazard ratio
HSD: honest significant difference
iTWINS: integrated covariates and Three‐Way INteraction Score
KEGG: Kyoto Encyclopedia of Genes and Genomes
LUAD: lung adenocarcinomas
LUSC: lung squamous cell carcinomas
NSCLC: non‐small‐cell lung cancer
QC: quality control
ROC: receiver operating characteristic
SD: standard deviation
SNP: single nucleotide polymorphisms
TCGA: The Cancer Genome Atlas
TIICs: tumor‐infiltrating immune cells
TIME: tumor immune microenvironment

## 1 Introduction

Lung cancer, as one of the most commonly diagnosed cancer, is the leading cause of cancer mortality worldwide, with an estimated 1.8 million deaths every year [1]. More than 85% of lung cancer cases are non‐small‐cell lung cancer (NSCLC), of which lung adenocarcinoma (LUAD) and lung squamous cell carcinoma (LUSC) are the two major histological subtypes [2, 3]. Despite recent advances in early diagnosis and therapy, the 5‐year survival rate is low, ranging from 4 to 17%, which depends on clinical characteristics [4]. Even with the same histological type and at the same clinical stage, there exists wide heterogeneity in overall survival because of varying therapeutic responses [5]. However, molecular mechanisms underlying these therapeutic responses remain largely unclear [6].

DNA methylation is a reversible epigenetic modification [7, 8], with aberrant methylation being an early precursor of cancer progression [9] and a possible therapeutic target for NSCLC [10, 11, 12, 13, 14, 15, 16, 17] and other cancers [18, 19]. Furthermore, epigenetic features are modifiable by various environmental exposures (e.g., cigarette smoking), and changes contribute to the development and progression of cancer as well. Cigarette smoking is one of the leading risk factors to lung cancer morbidity and mortality [20, 21]. Our previous study found that a two‐way interaction between cg05293407*_TRIM27_* and smoking reveals the histologically heterogeneous effect of *TRIM27* DNA methylation on survival among early‐stage NSCLC patients [22].

As is well known, gene–gene and gene–environment interactions provide important clues regarding biologic mechanisms of complex diseases [23] and are associated with the overall survival of NSCLC [10, 11, 14, 22, 24]. However, tumorigenesis and progression of complex diseases, such as NSCLC, often involves a multistep, multigenic, and multicausal biological process [25]; interactions between two relevant factors may only provide limited information understanding the cancer process driven by multiple factors [26]. Therefore, high‐order interactions may be needed to characterize complex association patterns relating to cancer recurrence and survival [27, 28], including NSCLC [29, 30]. This is because some well‐confirmed low‐order interactions, such as cg05293407*_TRIM27_* × smoking, may also be subjected to the influence of a third epigenetic factor, which necessitates the study of three‐way interactions arising from confirmed two‐way interactions [30]. To our knowledge, epigenetic analysis with three‐way interaction has seldom been conducted because of the complex nature of such analysis.

Hereby, we performed a two‐phase epigenome‐wide study of three‐way interactions on overall survival of early‐stage NSCLC based on the previously confirmed two‐way interaction between cg05293407*_TRIM27_* and smoking, using patients from international consortium and the Cancer Genome Atlas (TCGA) database.

## 2 Materials and methods

### 2.1 Study populations

Included in the study were early‐stage (stages I and II) NSCLC patients, with harmonized DNA methylation data and gene expression data, from five international study centers, including Harvard [31], Spain [32], Norway [33], and Sweden [34], and TCGA. All of these studies were approved by each Institutional Review Board, and patients provided written informed consent. The study methodologies conformed to the standards set by the Declaration of Helsinki and was approved by the local ethics committee.

#### 2.1.1 Harvard

The Harvard Lung Cancer Study cohort was described previously [31]. All patients were recruited at Massachusetts General Hospital since 1992. They were newly diagnosed and histologically confirmed as primary NSCLC at the time of recruitment. Snap‐frozen tumor samples were taken from patients during complete resection of the therapeutic procedure. Total 149 early‐stage patients, who had complete survival information, were enrolled in our study. Tumor DNA was extracted from 5‐μm‐thick histopathologic sections. Each specimen was evaluated by a pathologist for amount (tumor cellularity > 70%) and quality of tumor cells. Among them, 26 patients had mRNA sequencing data.

#### 2.1.2 Spain

As described previously [32], tumors were collected by surgical resection from 207 patients. DNA extraction was performed on tumor specimens (tumor cellularity > 50%). However, Spanish study center did not profile mRNA sequencing data.

#### 2.1.3 Norway

The Norwegian study consisted of 132 LUAD patients with operable lung cancer tumors seen at Oslo University Hospital, Rikshospitalet, Norway, in 2006–2011 [33]. Tumor tissues collected during surgery were snap‐frozen in liquid nitrogen and stored at −80 °C until DNA isolation. None of the enrolled patients received chemotherapy or radiotherapy before surgery. In addition, 93 of these patients had mRNA sequencing data.

#### 2.1.4 Sweden

Tumor tissue samples were collected from 36 patients with early‐stage NSCLC, at Skane University Hospital, Lund, Sweden [34]. The study was developed under the approval of the Regional Ethical Review Board in Lund, Sweden (Registration no. 2004/762 and 2008/702). Meanwhile, mRNA sequencing data were collected from 34 of these patients.

#### 2.1.5 TCGA

TCGA was composed of 227 LUAD and 241 LUSC cases with complete overall survival time and covariates. Level‐1 HumanMethylation450 DNA methylation data were downloaded at October 1, 2015. In addition, 221 LUAD and 235 LUSC patients had gene expression data. The TCGA workgroup completed processing and quality control (QC) of mRNA sequencing data. Raw counts were normalized by RNA‐seq expectation maximization (RSEM). Level‐3 gene quantification data were downloaded to further check quality. All of the gene expression value was transformed on a log_2_ scale and standardized before association analysis.

### 2.2 Quality control procedures for DNA methylation data

DNA methylation was profiled using Infinium HumanMethylation450 BeadChips (Illumina Inc., SanDiego, CA, USA). All studies followed the same QC procedures prior to association study. GenomeStudio Methylation Module V1.8 (Illumina Inc.) was used to convert raw image data into beta values (continuous numbers ranging from 0 to 100%) for background subtraction and control normalization. Unqualified probes meeting any one of the following criteria were excluded: (a) failed detection (*P* > 0.05) in more than 5% samples; (b) coefficient of variance (CV) < 5%; (c) methylated or unmethylated in all samples; (d) common single nucleotide polymorphisms (SNP) located in the probe sequence or 10‐bp flanking regions; (e) cross‐reactive probes or cross‐hybridizing probes [35]; or (f) did not pass quality control in all centers. Samples with > 5% undetectable probes or missing of pack‐year of smoking were excluded. Methylation signals were further processed for quantile normalization (*betaqn* function in R package *minfi*) as well as type I and II probes correction (*BMIQ* function in R package *lumi*), and were further adjusted for batch effects (*ComBat* function in R package *sva*) according to the best pipeline by a comparative study [36]. The QC processes are detailed in Fig. S1.

### 2.3 Statistical analysis

#### 2.3.1 A two‐phase study of three‐way interaction

**Fig. 1:**
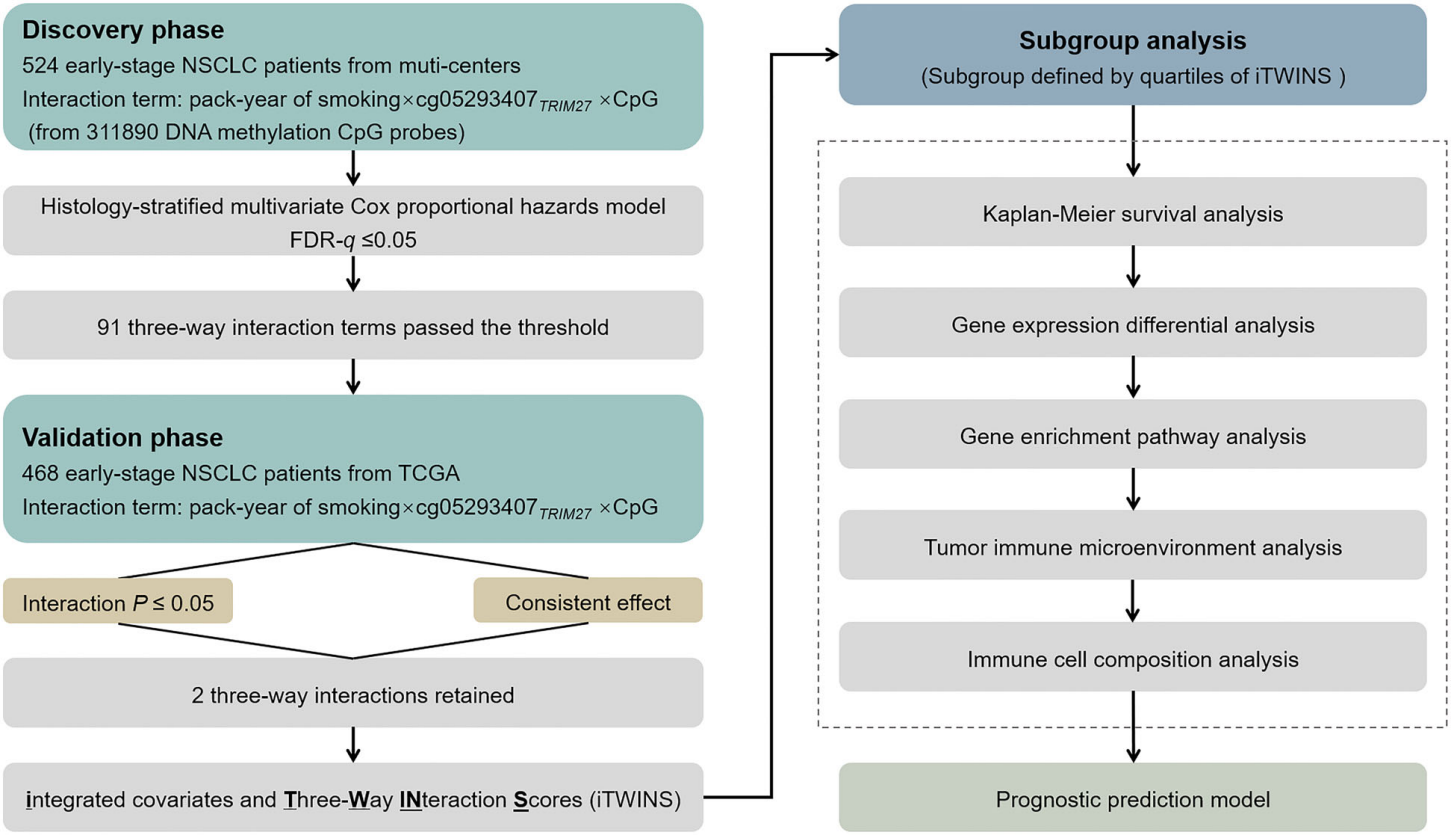
Flow chart of study design and statistical analyses. Patients with lung adenocarcinoma (LUAD) and lung squamous cell carcinoma (LUSC) from the Harvard, Spain, Norway, and Sweden cohorts were used in the discovery phase for screening, whereas data from the Cancer Genome Atlas (TCGA) were used for validation. Subgroup analyses were based on quartiles of iTWINS.

The analysis flow was depicted in Fig. 1, showing a two‐phase study to detect three‐way interactions that affect overall survival for early‐stage NSCLC on an epigenome‐wide scale. In the discovery phase, a histology‐stratified Cox proportional hazards model adjusted for age, sex, clinical stage, and study centers. The analysis flow was used to identify CpG probes that interact with pack‐year of smoking ×cg05293407*_TRIM27_*. That is, we tried to find three‐way interactions in the form of pack‐year of smoking × cg05293407*_TRIM27_* × CpG probe, by using samples from Harvard, Spain, Norway, and Sweden. Hazard ratios (HR) and 95% confidence intervals (CI) were computed for per 1% level of methylation increment. Multiple testing corrections were performed by controlling the false discovery rate (FDR) at a 5% level [37]. In the validation phase, the identified three‐way interactions from the discovery phase were further tested by using samples from TCGA. Significant interactions were those meeting the following criteria: (a) FDR*‐q* ≤ 0.05 in the discovery phase and *P* ≤ 0.05 in the validation phase; and (b) with consistent effect directions across two phases. Kaplan–Meier curves were used to compare survival differences between patients with high or low methylation level of each identified CpG probe, under different levels of smoking intensity and cg05293407*_TRIM27_* methylation.

#### 2.3.2 iTWINS subgroup analysis

An integrated covariates and Three‐Way INteraction Score (iTWINS) was defined by a weighted linear combination of all components inside the significant three‐way interaction, with weights derived from the aforementioned multivariable Cox proportional hazards model. Specifically,

$$\begin{array}{c} \text{iTWINS}=\sum \alpha_{i}\times {\text{Covariate}}_{i}+\beta_{1}\times \text{smoking}+\;\beta_{2}\times \text{cg}05293407_{TRIM\;27}+\\ \beta_{3}\times \text{CpG}+\;\beta_{12}\times \text{smoking}\times \text{cg}05293407_{TRIM\;27}+\;\beta_{13}\times \text{smoking}\times \text{CpG}+\\ \beta_{23}\times \;\text{cg}05293407_{TRIM\;27}\times \text{CpG}+\beta_{123}\times \;\text{smoking}\times \text{cg}05293407_{TRIM\;27}\times \text{CpG} \end{array}$$

where αs and βs were the coefficients estimated in the Cox model.

We conducted the following analyses. (a) We divided patients into four subgroups by the quartiles of iTWINS and compared their survival by Kaplan–Meier curves. (b) To explore the molecular characteristics of these subgroups, we preformed gene expression differential analysis through ANOVA (*aov* function in R package *multcomp*) and Tukey's honest significant difference (HSD) test (*TukeyHSD* function in R package *multcomp*). Genes with FDR‐*q* ≤ 0.05 and HSD‐*q* ≤ 0.05 were regarded as differentially expressed genes (DEGs). (c) We performed functional annotation and gene enrichment pathway analyses of Gene Ontology (GO) and Kyoto Encyclopedia of Genes and Genomes (KEGG) on the DEGs by using the *WebGestaltR* package in R [38, 39, 40]. FDR‐*q* ≤ 0.05 was considered statistically significant. (d) To explore the pattern of tumor immune microenvironment (TIME) among subgroups, we computed the immune score representing the level of infiltrating immune cells based on gene expression data by using the ESTIMATE algorithm [41]. (e) We used CIBERSORT, a deconvolution algorithm based on linear support vector regression, to quantify the compositions of 22 types of tumor‐infiltrating immune cells (TIICs) [42].

#### 2.3.3 Development of a prognostic prediction model

We further selected three‐way interactions by using a looser criterion (discovery: FDR‐*q* ≤ 0.10, validation: *P* ≤ 0.05). Then, they were included in a prognostic prediction model of NSCLC together with cg05293407*_TRIM27_* × pack‐year of smoking. The 3‐ and 5‐year overall survival of patients were estimated for each subgroup by using the Kaplan–Meier method [43]. The prediction accuracy was illustrated using a receiver operating characteristic (ROC) curve and was measured by area under the ROC curve (AUC) by the R package *survivalROC*. The 95% CI and *P* value of the AUC increase were calculated by 1000‐time bootstrap resampling. The concordance index (C‐index), an average accuracy of predictive survival across follow‐up years, which ranges from 0.5 to 1.0, were calculated to estimate the predictive performance.

Continuous variables were expressed as mean ± standard deviation (SD), and categorical variables were expressed in frequency (*n*) and proportion (%). Statistical analysis was performed using R version 3.6.1 (The R Foundation of Statistical Computing).

## 3 Results

After quality control, our epigenome‐wide DNA methylation data were composed of 311,891 CpG probes from 992 early‐stage NSCLC patients; 524 patients (*N*_LUAD_ = 425 and *N*_LUSC_ = 99) were in the discovery phase and 468 patients (*N*_LUAD_ = 227 and *N*_LUSC_ = 241) were in the validation phase. Demographic and clinical information for these patients were detailed in Table 1 and Table S1.

**Table 1:** Demographic and clinical descriptions for early‐stage NSCLC patients with DNA methylation data in five international study centers.

Variable	Discovery					Validation	Combined
	USA (*N* = 149)	Spain^a^ (*N* = 207)	Norway (*N* = 132)	Sweden (*N* = 36)	All (*N* = 524)	TCGA (*N* = 468)	Overall (*N* = 992)
Age (years)	67.78 ± 9.92	65.77 ± 10.66	65.4 ± 9.28	72.25 ± 7.22	66.7 ± 10.04	66.52 ± 9.3	66.61 ± 9.69
Pack‐year of smoking	51.49 ± 40.62	43.24 ± 31.72	26.93 ± 17.62	22.64 ± 25.19	40.06 ± 33.00	47.05 ± 28.40	43.36 ± 31.10
Sex							
Female	66 (44.30%)	99 (47.83%)	70 (53.03%)	22 (61.11%)	257 (49.05%)	185 (39.53%)	442 (44.56%)
Male	83 (55.70%)	108 (52.17%)	62 (46.97%)	14 (38.89%)	267 (50.95%)	283 (60.47%)	550 (55.44%)
Smoking status							
Never	18 (12.08%)	28 (13.53%)	16 (12.12%)	18 (50.00%)	80 (15.27%)	0 (0.00%)	80 (8.06%)
Former	81 (54.36%)	113 (54.59%)	74 (56.06%)	11 (30.56%)	279 (53.24%)	323 (69.02%)	602 (60.69%)
Current	50 (33.56%)	66 (31.88%)	42 (31.82%)	7 (19.44%)	165 (31.49%)	145 (30.98%)	310 (31.25%)
Clinical stage							
I	102 (68.46%)	167 (80.68%)	92 (69.70%)	34 (94.44%)	395 (75.38%)	301 (64.32%)	696 (70.16%)
II	47 (31.54%)	40 (19.32%)	40 (30.30%)	2 (5.56%)	129 (24.62%)	167 (35.68%)	296 (29.84%)
Histology							
LUAD	96 (64.43%)	169 (81.64%)	132 (100.00%)	28 (77.78%)	425 (81.11%)	227 (48.50%)	652 (65.73%)
LUSC	53 (35.57%)	38 (18.36%)	0 (0.00%)	8 (22.22%)	99 (18.89%)	241 (51.50%)	340 (34.27%)
Chemotherapy							
No	140 (93.96%)	166 (91.21%)	101 (76.52%)	25 (89.29%)	432 (87.98%)	151 (75.88%)	583 (84.49%)
Yes	9 (6.04%)	16 (8.79%)	31 (23.48%)	3 (10.71%)	59 (12.02%)	48 (24.12%)	107 (15.51%)
Unknown	0	25	0	8	33	269	302
Radiotherapy							
No	130 (87.25%)	172 (94.51%)	131 (99.24%)	28 (100.00%)	461 (93.89%)	190 (95.48%)	651 (94.35%)
Yes	19 (12.75%)	10 (5.49%)	1 (0.76%)	0 (0.00%)	30 (6.11%)	9 (4.52%)	39 (5.65%)
Unknown	0	25	0	8	33	269	302
Adjuvant therapy^b^							
No	125 (83.89%)	157 (86.26%)	100 (75.76%)	25 (89.29%)	407 (82.89%)	146 (73.37%)	553 (80.14%)
Yes	24 (16.11%)	25 (13.74%)	32 (24.24%)	3 (10.71%)	84 (17.11%)	53 (26.63%)	137 (19.86%)
Unknown	0	25	0	8	33	269	302
Survival year^c^							
Median (95% CI)	6.61 (5.14–7.49)	3.83 (3.21–4.46)	5.40 (5.16–5.77)	3.25 (2.09–4.39)	5.01 (4.53–5.25)	0.58 (0.50–0.70)	2.31 (2.00–2.64)
Censoring rate	18.79%	54.59%	68.94%	52.78%	47.90%	76.07%	61.19%

TCGA, The Cancer Genome Atlas; 95% CI, 95% confidence interval; LUAD, lung adenocarcinoma; LUSC, lung squamous cell carcinoma.

a Spain center is a collaborative study center, containing samples from Spain, Italy, the UK, France, and the USA.

b Adjuvant therapy included chemotherapy and/or radiotherapy.

c Restricted mean survival time was given because median was not available; proportion of samples lost to follow‐up or alive at the end of study.

### 3.1 Two significant three‐way interactions identified in the two‐phase study

In the discovery phase, 91 three‐way interactions (FDR‐*q* ≤ 0.05) were identified, while only 2 of them remained significant (*P* ≤ 0.05) in the validation phase and showed robust association in the combined data (Table 2). Two CpG probes, together with pack‐year of smoking and cg05293407*_TRIM27_*, had significant three‐way interaction effects on NSCLC survival, including cg00060500*_KIAA0226_* (*HR*_interaction_ = 0.993, 95% CI: 0.990‐0.996, *P* = 7.79 × 10^‐6^, FDR‐*q* = 0.039 in the discovery phase; *HR*_interaction_ = 0.992, 95% CI: 0.986‐0.999, *P* = 0.021 in the validation phase; *HR*_interaction_ = 0.993, 95% CI: 0.991‐0.996, *P* = 8.91 × 10^−7^ in the combined data) and cg17479956*_EXT2_* (*HR*_interaction_ = 0.997, 95% CI: 0.996‐0.998, *P* = 1.16 × 10^−5^, FDR‐*q* = 0.046 in the discovery phase; *HR*_interaction_ = 0.993, 95% CI: 0.987‐0.998, *P* = 0.011 in the validation phase; *HR*_interaction_ = 0.997, 95% CI: 0.996‐0.999, *P* = 5.72 × 10^‐5^ in the combined data); however, these two probes did not have significant marginal effects (Table S2) and were significantly associated with the expression of their corresponding genes (*KIAA0226* and *EXT2*), respectively (Fig. S2).

**Table 2:** Association results of two three‐way interactions in the discovery phase, the validation phase, and the combined data.

	Discovery phase					Validation phase				Combined data			
	HR	95% CI		*P*	FDR‐*q*	HR	95% CI		*P*	HR	95% CI		*P*
cg05293407	0.13	0.03	0.53	4.21E‐03		0.23	0.01	6.94	0.396	0.14	0.04	0.49	2.02E‐03
cg00060500	0.67	0.51	0.88	4.24E‐03		0.59	0.29	1.19	0.142	0.65	0.51	0.83	6.09E‐04
Pack‐year	0.90	0.86	0.94	2.06E‐06		0.88	0.80	0.97	0.012	0.90	0.86	0.93	1.04E‐07
cg05293407 × cg00060500	1.26	1.08	1.48	3.91E‐03		1.25	0.84	1.85	0.271	1.26	1.09	1.45	1.30E‐03
cg05293407 × pack‐year	1.07	1.04	1.10	2.32E‐06		1.07	1.01	1.14	0.016	1.07	1.04	1..09	1.14E‐07
cg00060500 × pack‐year	1.01	1.01	1.02	4.23E‐06		1.01	1.00	1.03	0.019	1.01	1.01	1.02	6.62E‐07
cg05293407 × cg00060500 × pack‐year	0.993	0.990	0.996	7.79E‐06	0.039	0.992	0.986	0.999	0.021	0.993	0.991	0.996	8.91E‐07
cg05293407	0.22	0.10	0.46	4.97E‐05		0.20	0.02	1.76	0.146	0.32	0.17	0.61	5.23E‐04
cg17479956	0.73	0.64	0.83	2.57E‐06		0.63	0.41	0.97	0.034	0.77	0.68	0.87	4.12E‐05
pack‐year	0.95	0.93	0.97	1.37E‐05		0.91	0.85	0.99	0.019	0.95	0.93	0.97	1.74E‐05
cg05293407 × cg17479956	1.18	1.10	1.27	1.94E‐06		1.32	1.03	1.70	0.031	1.14	1.07	1.22	4.18E‐05
cg05293407 × pack‐year	1.03	1.02	1.04	3.15E‐06		1.06	1.01	1.11	0.015	1.03	1.02	1.04	4.69E‐06
cg17479956 × pack‐year	1.005	1.003	1.008	2.46E‐05		1.011	1.002	1.019	0.017	1.005	1.003	1.007	8.99E‐05
cg05293407 × cg17479956 × pack‐year	0.997	0.996	0.998	1.16E‐05	0.046	0.993	0.987	0.998	0.011	0.997	0.996	0.999	5.72E‐05

**Fig. 2:**
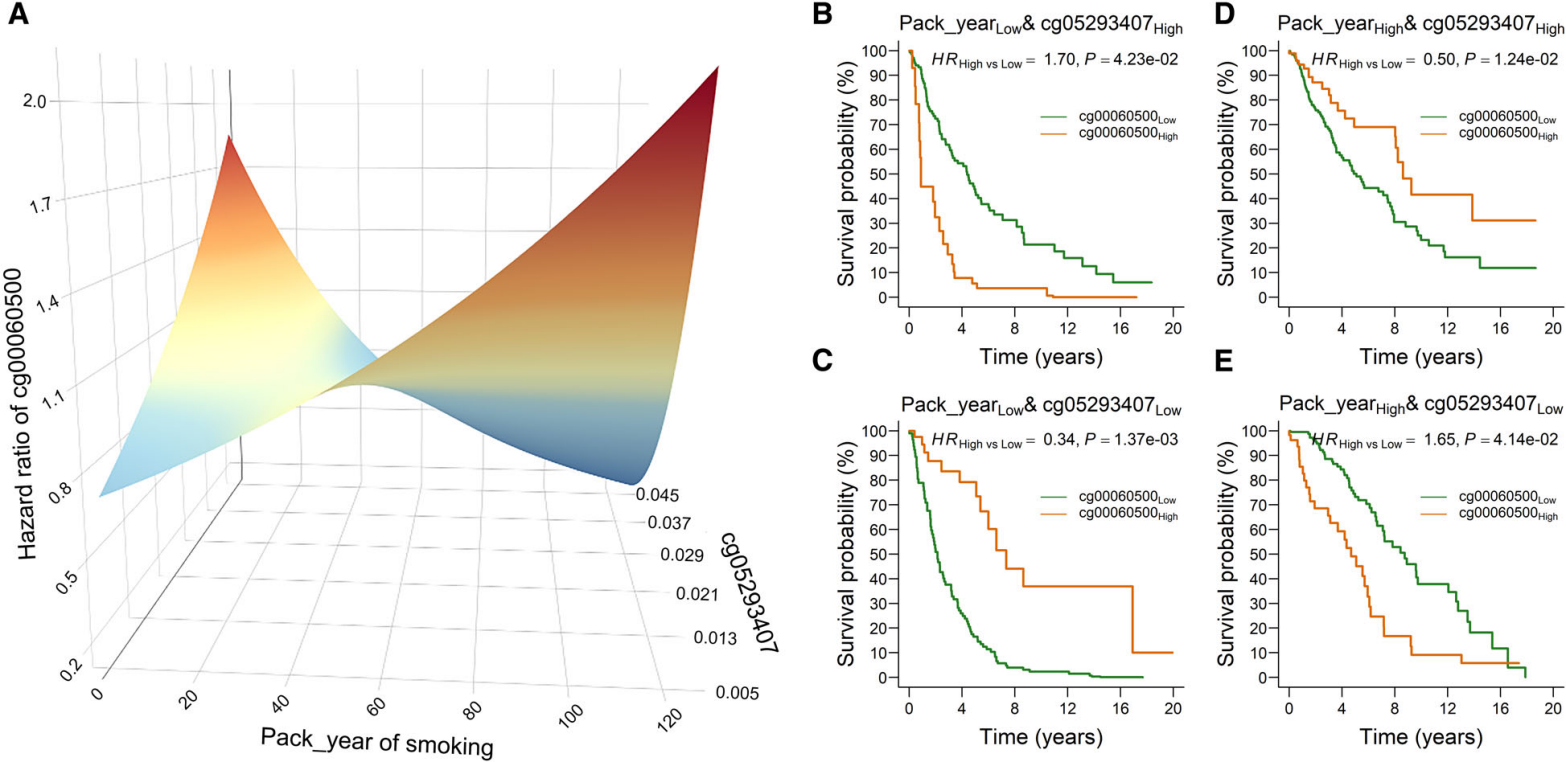
The three‐way interaction pattern between pack‐year of smoking, cg05293407*_TRIM27_* and cg00060500*_KIAA0226_*. (A) The pattern illustrated by a 3D plot. (B‐E) Kaplan–Meier survival analysis of cg00060500*_KIAA0226_* stratified by pack‐year of smoking and cg05293407*_TRIM27_*. The number of patients in each subgroup was 266, 282, 211 and 233. Hazard ratio (HR), 95% CI, and *P*‐values were derived from a Cox proportional hazards regression model adjusted for age, sex, clinical stage and study centers.

To visualize the three‐way interactions, we generated a 3D figure illustrating, for example, the varying effects of cg00060500*_KIAA0226_* at different levels of pack‐year of smoking and cg05293407*_TRIM27_* (Fig. 2A). As the smoking intensity increased and methylation level of cg05293407*_TRIM27_* decreased, we observed an enhanced risk associated with cg00060500*_KIAA0226_*; the risk of cg00060500*_KIAA0226_* was elevated as smoking intensity decreased and methylation level of cg05293407*_TRIM27_* increased.

To more clearly illustrate the effect modifiers, we compared the effect of cg00060500*_KIAA0226_* (high vs low defined by median) on survival in four subgroups, defined by the median of pack‐year of smoking and cg05293407*_TRIM27_* (Fig. 2B‐E). For patients with low level of pack‐year of smoking, the effect of cg00060500*_KIAA0226_* on survival was harmful (*HR*_High vs Low_ = 1.70, 95% CI: 1.02‐2.83, *P* = 0.042) when cg05293407*_TRIM27_* was high (Fig. 2B), and was protective (*HR*_High vs Low_ = 0.34, 95% CI: 0.17‐0.66, *P* = 1.37 × 10^‐3^) when cg05293407*_TRIM27_* was low (Fig. 2C); for patients with high level of pack‐year of smoking, cg00060500*_KIAA0226_* had a protective effect (*HR*_High vs Low_ = 0.50, 95% CI: 0.29‐0.86, *P* = 0.012) when cg05293407*_TRIM27_* was high (Fig. 2D), and a detrimental effect (*HR*_High vs Low_ = 1.65, 95% CI: 1.02‐2.68, *P* = 0.041) when cg05293407*_TRIM27_* was low (Fig. 2E). Similar patterns were also observed for the three‐way interaction of pack‐year of smoking × cg05293407*_TRIM27_* × cg17479956*_EXT2_* (Fig. S3).

As demonstrated in Fig. S4A, these two three‐way interaction modules were highly correlated (*r* = 0.82, *P* = 2.66 × 10^‐241^), and *KIAA0226* and *EXT2* gene expression were also correlated significantly (*r* = 0.61, *P* = 7.71 × 10^‐52^) (Fig. S4B). Therefore, the subsequent analysis focused on the interaction involving cg00060500*_KIAA0226_* that had a lower *P* value.

### 3.2 Significant heterogeneity among iTWINS subgroups

**Fig. 3:**
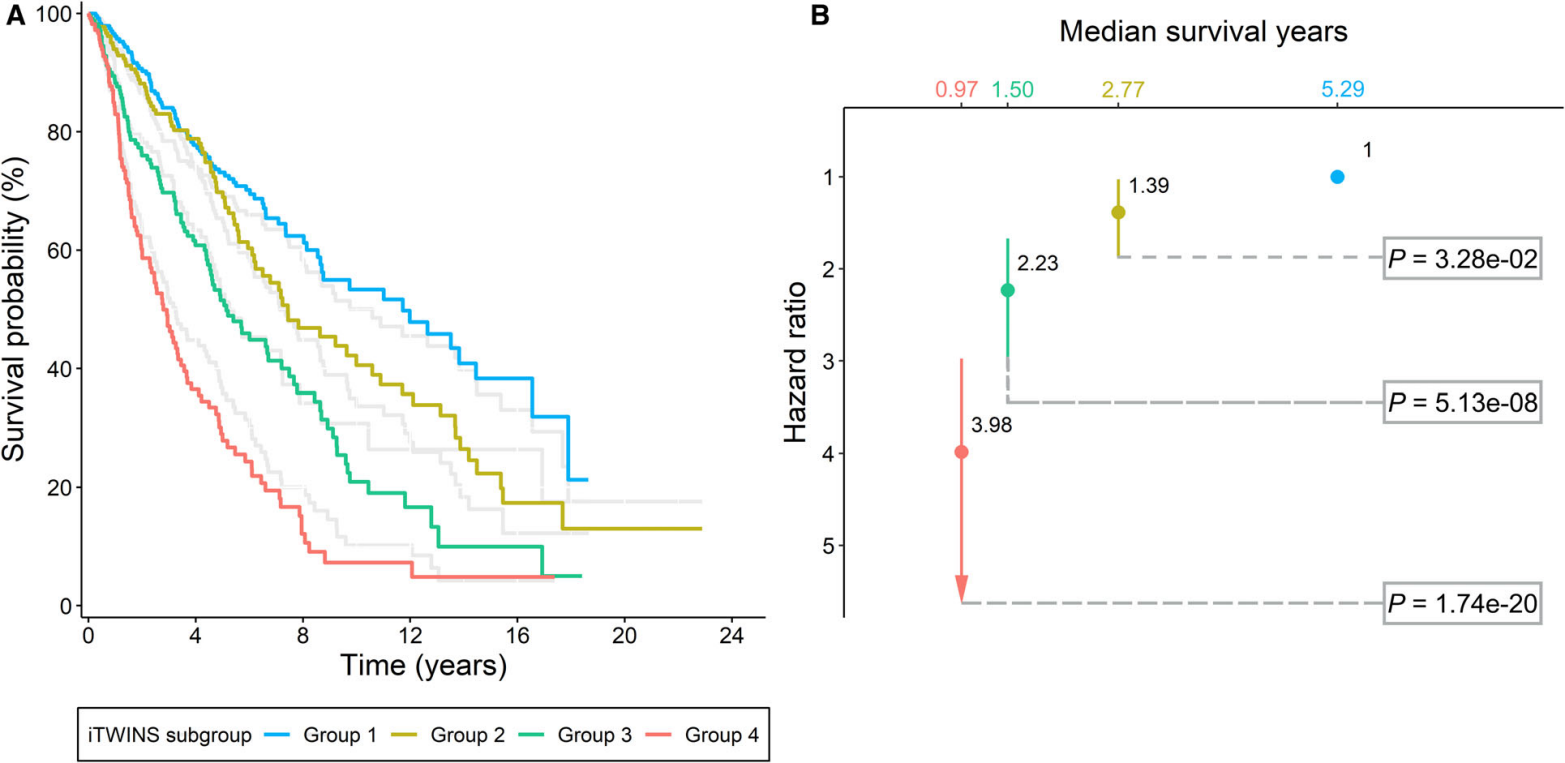
Estimated survival curves for patients among iTWINS subgroups. (A) Kaplan–Meier survival curves for patients grouped by iTWINS subgroups. Patients were categorized into four subgroups by using the quantile of iTWINS as the cutoffs. The number of patients in groups 1‐4 was 248. (B) Hazard ratio (HR) and *P*‐values were derived from the Cox proportional hazards model for patients with different quartile levels of the iTWINS.

**Fig. 4:**
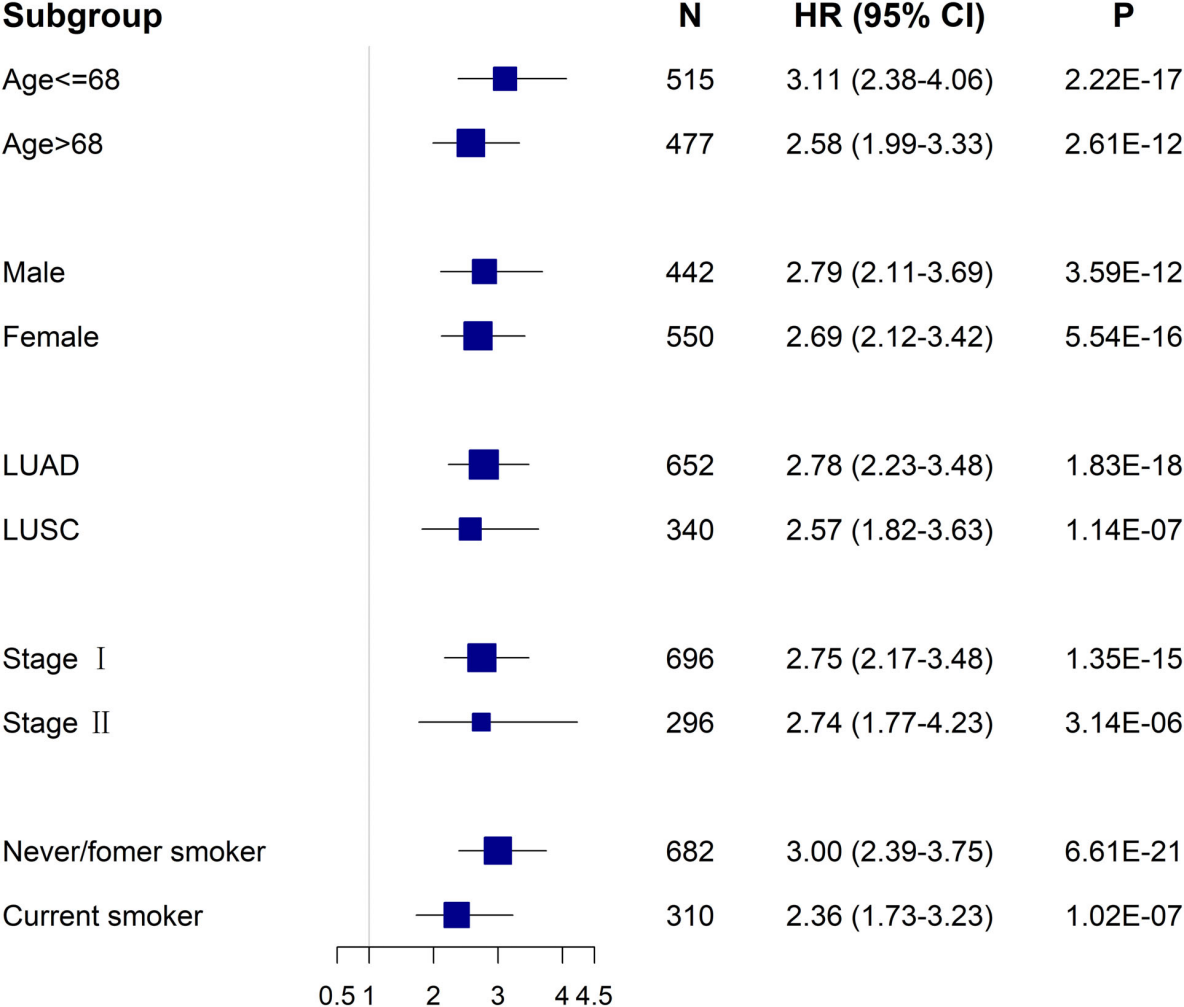
Forest plots of results from stratification analysis of iTWINS. Hazard ratio (HR) with 95% CI of iTWINS on non‐small‐cell lung cancer (NSCLC) survival in various subgroups is stratified by clinical characteristics. LUAD, lung adenocarcinoma; LUSC, lung squamous cell carcinoma. Hazard ratio (HR), 95% CI, and *P*‐values were derived from a Cox proportional hazards regression model for patients in different subgroups.

We categorized patients into four subgroups based on the quartiles of iTWINS, with Groups 1 and 4 representing the lowest and highest risk patients, respectively. As shown in Fig. 3A, patients with higher iTWINS had a shorter median survival year (0.97, 95% CI: 0.75–1.25) and a higher risk of mortality (*HR*_Group4 vs 1_ = 3.98, 95% CI: 2.98–5.33, *P* = 1.74 × 10^‐20^; *HR*_Group3 vs 1_ = 2.23, 95% CI: 1.67–2.98, *P* = 5.13 × 10^‐8^; *HR*_Group2 vs 1_ = 1.39, 95% CI: 1.03–1.87, *P* = 0.033) (Fig. 3B). For comparison, we also divided patients into four subgroups based on the clinical score, a weighted linear combination of demographic and clinical variables, as shown in gray lines in Fig. 3A. iTWINS obviously outperformed the clinical score in discriminability, and the association between iTWINS and overall survival remained significant in all subgroup analyses stratified by various covariates (Fig. 4).

**Fig. 5:**
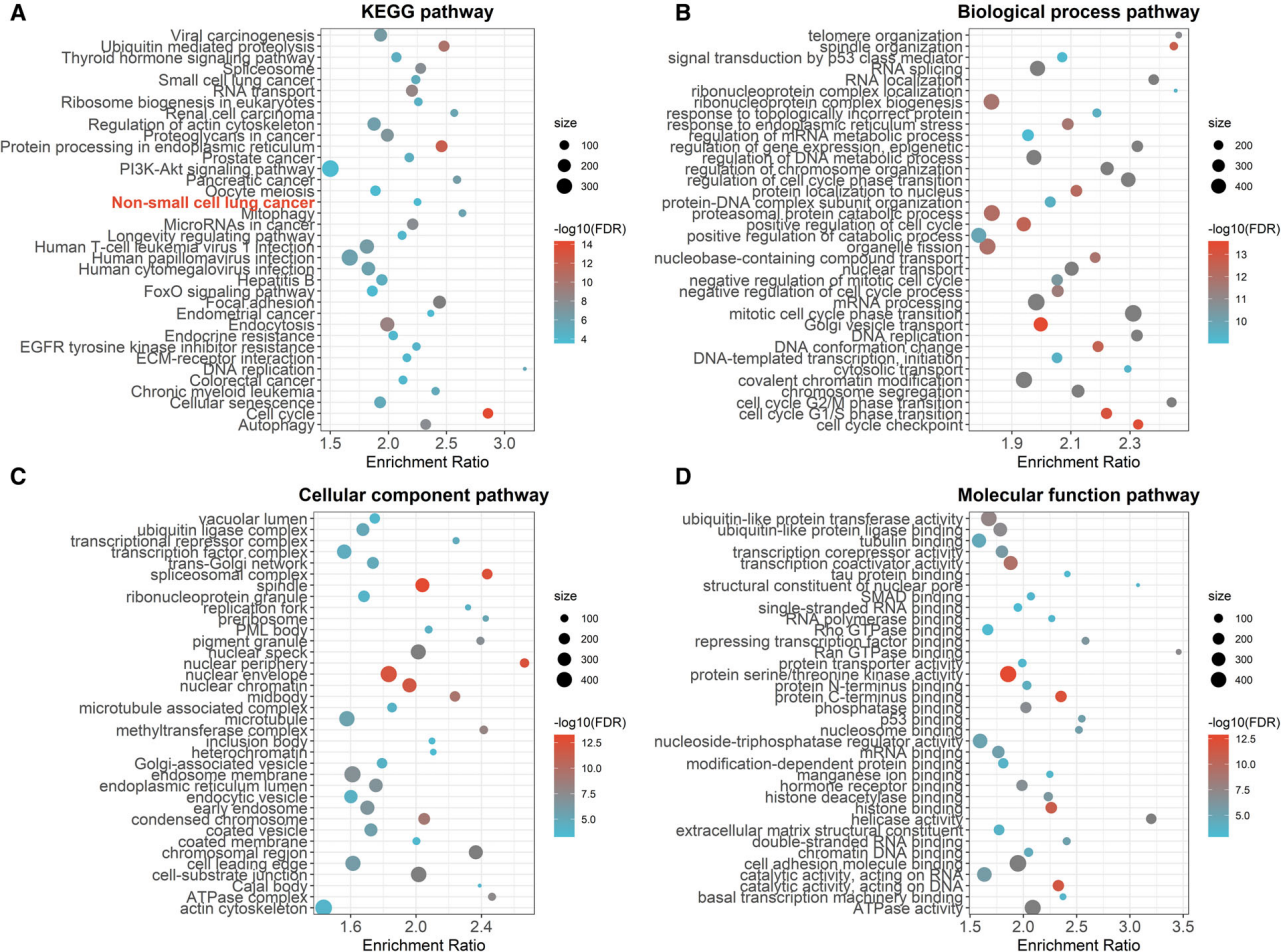
The functional enrichment analyses of DEGs. (A) Top 36 significant KEGG pathways. (B) Top 36 significant biological process pathways. (C) Top 36 significant cellular component pathways and (D) top 36 significant molecular function pathways.

**Fig. 6:**
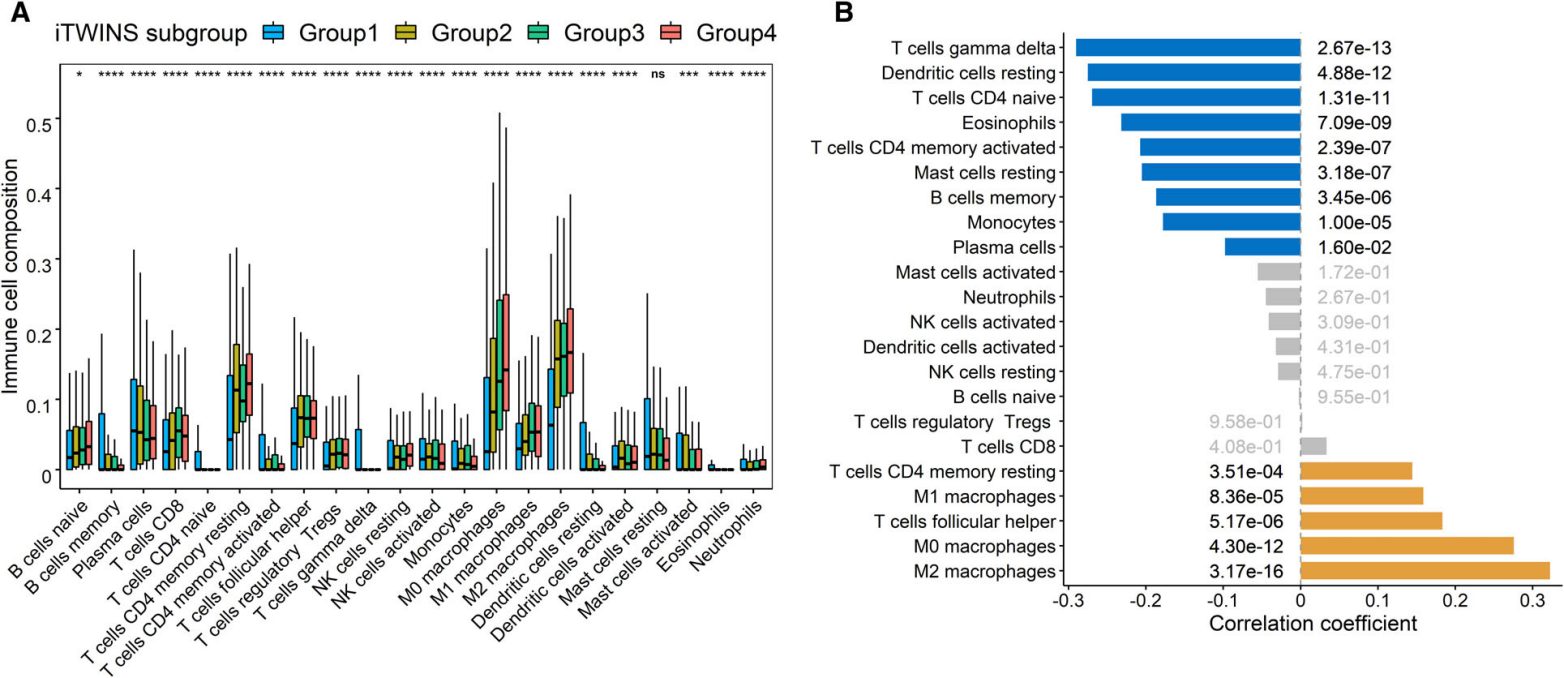
The association analysis between immune cells and iTWINS. (A) Comparisons of the abundances of 22 immune cells in iTWINS subgroups. ‘ns’ means *P* > 0.05, ∗ means *P* < 0.05, ∗∗∗ means *P* < 0.001 and ∗∗∗∗ means *P* < 0.0001. (B) Correlation analysis between immune cells and iTWINS. Yellow bars meant correlation coefficient > 0 and *P* ≤ 0.05, blue bars meant correlation coefficient< 0 and *P* ≤ 0.05, and gray bars meant *P* > 0.05. Correlation coefficients and hypothesis tests were based on Pearson correlation tests.

Exploring the molecular characteristics of these iTWINS subgroups, we performed differential expression analysis and identified a total of 3150 DEGs, which were significantly enriched in 106 KEGG pathways, including NSCLC (Fig. 5A). In addition, GO enrichment analysis identified 284 significant biological process pathways (Fig. 5B), 76 significant cellular component pathways (Fig. 5C) and 90 significant molecular function pathways (Fig. 5D). Moreover, iTWINS was significantly but negatively correlated with the immune score (*r* = −0.096, *P* = 0.018) (Fig. S5A). For example, compared to Group 4 of iTWINS that had the worst survival, Group 1 with the best survival had the highest immune score (*P* = 0.0092) (Fig. S5B), indicating that abundant immune cells are beneficial for NSCLC survival. Further, the compositions of 14 types of immune cells were differently distributed among iTWINS subgroups (Fig. 6A); the correlation of iTWINS with each immune cell composition varied, including negative (e.g., with T‐cell subsets gamma delta, *r* = −0.29, *P* = 2.67 × 10^‐13^) and positive correlations (e.g., with M2 macrophages, *r* = 0.32, *P* = 3.17 × 10^‐16^) (Fig. 6B).

### 3.3 Three‐way interaction empowered prognostic prediction model

**Fig. 7:**
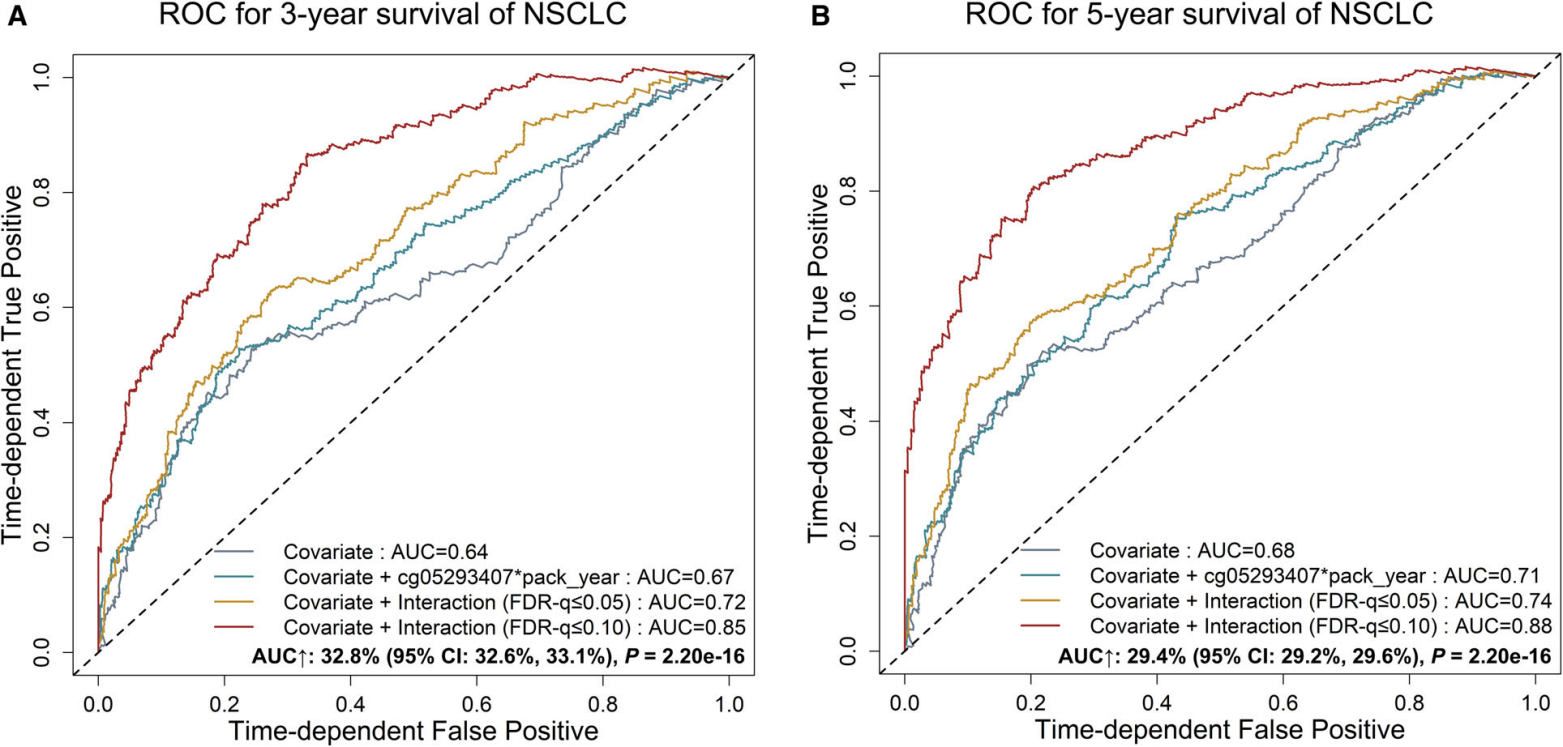
ROC curves for different predictive models using the covariates (clinical information), two‐way interaction (cg05293407*_TRIM27_* and pack‐year of smoking) and three‐way interactions (cg05293407*_TRIM27_*, pack‐year of smoking and another CpG probe) with FDR‐*q* ≤ 0.05 or FDR‐*q* ≤ 0.10. (A) Three‐year survival prediction. (B) Five‐year survival prediction. The AUC increase (%) was evaluated by comparing the model with that with only the covariates. *P*‐values and 95% CIs were calculated by using 1000 bootstrap samples and t‐tests. AUC, area under the receiver operating characteristic curve; ROC, receiver operating characteristic.

We built a prognostic prediction model of NSCLC by incorporating significant three‐way interactions. The model with only demographic and clinical variables had a limited prediction ability (AUC_3‐year_ = 0.64, AUC_5‐year_ = 0.68, C‐index = 0.62), and the prediction accuracy slightly increased by adding the cg05293407*_TRIM27_* × pack‐year of smoking (AUC_3‐year_ = 0.67, AUC_5‐year_ = 0.71, C‐index = 0.63) or the two significant three‐way interactions (AUC_3‐year_ = 0.72, AUC_5‐year_ = 0.74, C‐index = 0.66). However, by adding 42 three‐way interactions obtained by using a looser criterion (discovery: FDR‐*q* ≤ 0.10, validation: *P* ≤ 0.05), the AUC increased by 32.8% (95% CI: 32.6%‐33.1%, *P* = 2.20 × 10^‐16^) and 29.4% (95% CI: 29.2%‐29.6%, *P* = 2.20 × 10^‐16^) for 3‐ and 5‐year survival (AUC_3‐year_ = 0.85, AUC_5‐year_ = 0.88, C‐index = 0.76), respectively (Fig. 7).

## 4 Discussion

We were among the first few to perform an epigenome‐wide three‐way interaction study on overall survival of early‐stage NSCLC. We identified two CpG probes (cg00060500*_KIAA0226_* and cg17479956*_EXT2_*), each of which, by interacting with cg05293407*_TRIM27_* × pack‐year of smoking, displayed a significant three‐way interaction effect on the overall survival of NSCLC. The proposed iTWINS had some interesting properties. It enabled us to categorize patients with various risk levels of mortality into four subgroups, which involved a series of significant DEGs belonging to several biological pathways. Also, iTWINS was negatively correlated with the immune score, representing the level of infiltrating immune cells. Finally, prognostic models with three‐way interactions substantially improved the prediction accuracy.

It is known that gene–gene and gene–environment interactions on an epigenetic level play a crucial role in revealing biological mechanisms of complex diseases [23], including NSCLC [11, 24]. Emerging evidence from higher‐order interaction studies with high‐throughput data [44, 45] indicates that accommodation of such complex association patterns can improve the predictive power [46]. Building upon our previous study that identified two‐way interactions contributing to the improvement of the prediction accuracy [14], the current study found that inclusion of three‐way interactions might largely enhance prediction performance for both 3‐ and 5‐year survival. Therefore, complex association patterns (e.g., higher‐order interactions) among multiple factors should be factored in for studies of complex diseases, such as NSCLC.

To further elaborate on the three‐way interactions among pack‐year of smoking, cg05293407*_TRIM27_* and cg00060500*_KIAA0226_*/cg17479956*_EXT2_*, we generated 3D figures and observed varying effects of cg00060500*_KIAA0226_*/cg17479956*_EXT2_* at different levels of pack‐year of smoking and cg05293407*_TRIM27_*. Nevertheless, *EXT2* is found to be the likely antigens in the immune complexes and is possibly the target antigens in autoimmune membranous nephropathy [47]. Meanwhile, *KIAA0226* is identified as a multifaceted immune modulatory protein that regulates autophagy and phagocytosis [48]. They were all related to immunity, and their gene expression was significantly correlated. Thus, the subsequent analysis only focused on the interaction involving cg00060500*_KIAA0226_*.

Biologically, *KIAA0226* was identified as a novel *Beclin‐1*‐binding protein, and hence dubbed as *Rubicon* (RUN domain and cysteine‐rich domain containing, *Beclin‐1*‐interacting protein) [49, 50]. *Rubicon* is a multifaceted immune modulatory protein that regulates autophagy and phagocytosis [48]. Cigarette smoke induced extrinsic apoptosis partly dependent on autophagy protein *Beclin‐1* and caused a reduction of *Beclin‐1* expression [51, 52]. Furthermore, decreased expression of *Beclin‐1* may lead to changes in *Rubicon* expression, thus affecting cross‐regulation between apoptosis and autophagy [53]. That said, biological mechanisms underlying this three‐way interaction need to be more systematically studied.

Notably, besides the heterogeneous survival among four epigenetic iTWINS subgroups, there were a series of significant transcriptional DEGs enriched in cancer related pathways, including NSCLC pathway and EGFR tyrosine kinase inhibitor resistance pathway which directly affected the overall survival of NSCLC [54]. In addition, the subgroup with the lowest iTWINS had the best survival and the highest immune score, consistent with the previous study [55]. Furthermore, among 13 immune cell types identified in NSCLC tumors [56], 7 of them were significantly correlated with iTWINS in our study, including T cells CD4, T cells CD8, B cells, macrophages, eosinophils, mast cells, and dendritic cells. For example, M2 macrophages were positively correlated with iTWINS, indicating the higher abundance of M2 macrophages, the worse the survival of NSCLC, as confirmed by several studies [57, 58]. Our iTWINS subgroup analysis further indicated that potential NSCLC‐related pathways and TIICs might be the driven factors of lung cancer progression.

Our study has several strengths. First, to our knowledge, this is the first three‐way interaction study between DNA methylation of CpG probes and pack‐year of smoking, shedding some light on the tumor recurrence and progression. Second, we controlled false positives in the discovery phase and also performed external validation using an independent population. In our study, the final significant signals were defined as these with FDR‐*q* ≤ 0.05 in the discovery phase and meanwhile with *P* ≤ 0.05 in the validation phase. Thus, the overall false positive rate is less than 0.05, which is approximatively around 0.0025 (0.05 × 0.05). Such two‐phase design is a very popular and quite conservative manner used in the omics analysis to control false positives. Third, 3D plots were designed and used to illustrate the complex three‐way interactions, facilitating interpretation and dissemination of our results. Fourth, by exploring the molecular characteristics of different subgroups defined by iTWINS, we gained a better understanding of the heterogeneous response to immunotherapy, and therefore, a potential cause of survival heterogeneity. Finally, our prognostic model by incorporating three‐way interactions may aid physicians in making clinical decisions or guiding immunotherapy.

We acknowledge limitations. First, the study did not elucidate the biological mechanism underlying the identified three‐way interactions, although the results of immune‐related and enrichment analyses might provide insight into the functional mechanisms. Second, the censoring rate (76.07%) of survival time for the TCGA cohort is relatively high, since early‐stage NSCLC patients need longer follow‐up time. Thus, the validation phase using TCGA population had low statistical power. Nonetheless, the three‐way interactions remained significant in TCGA. Finally, as the majority of our population was Caucasian (89%), generalization of the results to the other ethnicity groups should be cautioned.

## 5 Conclusion

Our study identified two CpG probes (cg00060500*_KIAA0226_* and cg17479956*_EXT2_*), together with pack‐year of smoking and cg05293407*_TRIM27_*, had genome‐wide significant three‐way interaction effects on NSCLC survival. iTWINS, which involved in TIME, can distinguish high risk NSCLC patients susceptible to mortality, by significantly improving prognostic prediction accuracy for NSCLC survival. Our findings suggested that lung cancer progression may be driven by complex interacting effects that involve multiple environmental and epigenetic factors, which can provide potential therapeutic targets of NSCLC.

## Conflict of interest

The authors declare no conflict of interest.

### Peer review

The peer review history for this article is available at https://publons.com/publon/10.1002/1878‐0261.13167.

## Author contributions

XJ, LL, JF, RZ, FC, and DCC contributed to the study design. RZ, LS, AS, MMB, AK, MP, JS, ÅH, ME, and DCC contributed to data collection. XJ, LL, JF, YL, YW, SS, RZ, FC, and DCC performed statistical analysis and interpretation and drafted the manuscript. XJ, LL, JF, YL, YW, and SS revised the manuscript. All authors contributed to critical revision of the manuscript and approved its final version. Financial support and study supervision were provided by RZ, FC, and DCC.

## Consent for publication

All participants or their surrogate care providers gave written informed consent. All authors have reviewed the manuscript and consented for publication.

## Web resources

TCGA: https://portal.gdc.cancer.gov

## Supporting information


**Table S1**. Demographic and clinical descriptions of early‐stage NSCLC patients with gene expression data in four international study centers.
**Table S2**. The association results of variables derived from Cox proportional hazards model adjusted for covariates in NSCLC samples.
**Fig. S1**. Quality control processes for DNA methylation data.
**Fig. S2**. Correlation between DNA methylation probe and its corresponding gene expression.
**Fig. S3**. The three‐way interaction pattern between pack‐year of smoking, cg05293407*_TRIM27_* and cg17479956*_EXT2_*.
**Fig. S4**. Association between *KIAA0226* and *EXT2*.
**Fig. S5**. The association analysis between immune score and iTWINS.

## Data Availability

The DNA methylation image data of Harvard, Spain, Norway, and Sweden study cohort can be requested from DCC, ME, ÅH, and JS, respectively. Alternatively, it can be retrieved from gene expression omnibus database (GSE39279, GSE66836 and GSE56044). TCGA: https://tcga‐data.nci.nih.gov; now hosted at GDC: https://portal.gdc.cancer.gov.

## References

[1] Sung H , Ferlay J , Siegel RL , Laversanne M , Soerjomataram I , Jemal A , et al. Global Cancer Statistics 2020: GLOBOCAN estimates of incidence and mortality worldwide for 36 cancers in 185 countries. CA Cancer J Clin. 2021;71:209–49. 33538338 10.3322/caac.21660

[2] Chen Z , Fillmore CM , Hammerman PS , Kim CF , Wong KK . Non‐small‐cell lung cancers: a heterogeneous set of diseases. Nat Rev Cancer. 2014;14:535–46. 25056707 10.1038/nrc3775 PMC5712844

[3] Molina JR , Yang P , Cassivi SD , Schild SE , Adjei AA . Non‐small cell lung cancer: epidemiology, risk factors, treatment, and survivorship. Mayo Clin Proc. 2008;83:584–94. 18452692 10.4065/83.5.584 PMC2718421

[4] Hirsch FR , Scagliotti GV , Mulshine JL , Kwon R , Curran WJ Jr , Wu YL , et al. Lung cancer: current therapies and new targeted treatments. Lancet. 2017;389:299–311. 27574741 10.1016/S0140-6736(16)30958-8

[5] Zito Marino F , Bianco R , Accardo M , Ronchi A , Cozzolino I , Morgillo F , et al. Molecular heterogeneity in lung cancer: from mechanisms of origin to clinical implications. Int J Med Sci. 2019;16:981–9. 31341411 10.7150/ijms.34739 PMC6643125

[6] Tang S , Pan Y , Wang Y , Hu L , Cao S , Chu M , et al. Genome‐wide association study of survival in early‐stage non‐small cell lung cancer. Ann Surg Oncol. 2015;22:630–5. 25145502 10.1245/s10434-014-3983-0

[7] Egger G , Liang G , Aparicio A , Jones PA . Epigenetics in human disease and prospects for epigenetic therapy. Nature. 2004;429:457–63. 15164071 10.1038/nature02625

[8] Feinberg AP , Tycko B . The history of cancer epigenetics. Nat Rev Cancer. 2004;4:143–53. 14732866 10.1038/nrc1279

[9] Heyn H , Esteller M . DNA methylation profiling in the clinic: applications and challenges. Nat Rev Genet. 2012;13:679–92. 22945394 10.1038/nrg3270

[10] Zhang R , Lai L , He J , Chen C , You D , Duan W , et al. EGLN2 DNA methylation and expression interact with HIF1A to affect survival of early‐stage NSCLC. Epigenetics. 2019;14:118–29. 30665327 10.1080/15592294.2019.1573066 PMC6557590

[11] Zhang R , Lai L , Dong X , He J , You D , Chen C , et al. SIPA1L3 methylation modifies the benefit of smoking cessation on lung adenocarcinoma survival: an epigenomic‐smoking interaction analysis. Mol Oncol. 2019;13:1235–48. 30924596 10.1002/1878-0261.12482 PMC6487703

[12] Dong X , Zhang R , He J , Lai L , Alolga RN , Shen S , et al. Trans‐omics biomarker model improves prognostic prediction accuracy for early‐stage lung adenocarcinoma. Aging. 2019;11:6312–35. 31434796 10.18632/aging.102189 PMC6738411

[13] Wei Y , Liang J , Zhang R , Guo Y , Shen S , Su L , et al. Epigenetic modifications in KDM lysine demethylases associate with survival of early‐stage NSCLC. Clin Epigenetics. 2018;10:41. 29619118 10.1186/s13148-018-0474-3 PMC5879927

[14] Zhang R , Chen C , Dong X , Shen S , Lai L , He J , et al. Independent validation of early‐stage NSCLC prognostic scores incorporating epigenetic and transcriptional biomarkers with gene‐gene interactions and main effects. Chest. 2020;158:808. 10.1016/j.chest.2020.01.048. 32113923 PMC7417380

[15] Guo Y , Zhang R , Shen S , Wei Y , Salama SM , Fleischer T , et al. DNA Methylation of LRRC3B: a biomarker for survival of early‐stage non‐small cell lung cancer patients. Cancer Epidemiol Biomarkers Prev. 2018;27:1527–35. 30185536 10.1158/1055-9965.EPI-18-0454 PMC6279565

[16] Shen S , Zhang R , Guo Y , Loehrer E , Wei Y , Zhu Y , et al. A multi‐omic study reveals BTG2 as a reliable prognostic marker for early‐stage non‐small cell lung cancer. Mol Oncol. 2018;12:913–24. 29656435 10.1002/1878-0261.12204 PMC5983115

[17] Shen S , Wei Y , Zhang R , Du M , Duan W , Yang S , et al. Mutant‐allele fraction heterogeneity is associated with non‐small cell lung cancer patient survival. Oncol Lett. 2018;15:795–802. 29399148 10.3892/ol.2017.7428 PMC5772758

[18] Joo JE , Dowty JG , Milne RL , Wong EM , Dugue PA , English D , et al. Heritable DNA methylation marks associated with susceptibility to breast cancer. Nat Commun. 2018;9:867. 29491469 10.1038/s41467-018-03058-6 PMC5830448

[19] Widschwendter M , Zikan M , Wahl B , Lempiäinen H , Paprotka T , Evans I , et al. The potential of circulating tumor DNA methylation analysis for the early detection and management of ovarian cancer. Genome Med. 2017;9:116. 29268796 10.1186/s13073-017-0500-7 PMC5740748

[20] Flanders WD , Lally CA , Zhu BP , Henley SJ , Thun MJ . Lung cancer mortality in relation to age, duration of smoking, and daily cigarette consumption: results from Cancer Prevention Study II. Cancer Res. 2003;63:6556–62. 14559851

[21] Janjigian YY , McDonnell K , Kris MG , Shen R , Sima CS , Bach PB , et al. Pack‐years of cigarette smoking as a prognostic factor in patients with stage IIIB/IV nonsmall cell lung cancer. Cancer. 2010;116:670–5. 20029977 10.1002/cncr.24813 PMC2815173

[22] Ji X , Lin L , Shen S , Dong X , Chen C , Li Y , et al. Epigenetic‐smoking interaction reveals histologically heterogeneous effects of TRIM27 DNA methylation on overall survival among early‐stage NSCLC patients. Mol Oncol. 2020;14:2759–74. 33448640 10.1002/1878-0261.12785 PMC7607178

[23] Trerotola M , Relli V , Simeone P , Alberti S . Epigenetic inheritance and the missing heritability. Hum Genomics. 2015;9:17. 26216216 10.1186/s40246-015-0041-3 PMC4517414

[24] Chen C , Wei Y , Wei L , Chen J , Chen X , Dong X , et al. Epigenome‐wide gene‐age interaction analysis reveals reversed effects of PRODH DNA methylation on survival between young and elderly early‐stage NSCLC patients. Aging. 2020;12:10642–62. 32511103 10.18632/aging.103284 PMC7346054

[25] Mucci LA , Wedren S , Tamimi RM , Trichopoulos D , Adami HO . The role of gene‐environment interaction in the aetiology of human cancer: examples from cancers of the large bowel, lung and breast. J Intern Med. 2001;249:477–93. 11422654 10.1046/j.1365-2796.2001.00839.x

[26] Kraft P , Hunter DJ . Genetic risk prediction–are we there yet? N Engl J Med. 2009;360:1701–3. 19369656 10.1056/NEJMp0810107

[27] Tekin E , White C , Kang TM , Singh N , Cruz‐Loya M , Damoiseaux R , et al. Prevalence and patterns of higher‐order drug interactions in Escherichia coli. NPJ Syst Biol Appl. 2018;4:31. 30181902 10.1038/s41540-018-0069-9 PMC6119685

[28] Basu S , Kumbier K , Brown JB , Yu B . Iterative random forests to discover predictive and stable high‐order interactions. Proc Natl Acad Sci USA. 2018;115:1943–8. 29351989 10.1073/pnas.1711236115 PMC5828575

[29] Woods NT , Monteiro AN , Thompson ZJ , Amankwah EK , Naas N , Haura EB , et al. Interleukin polymorphisms associated with overall survival, disease‐free survival, and recurrence in non‐small cell lung cancer patients. Mol Carcinog. 2015;54(Suppl 1):E172–84. 25597281 10.1002/mc.22275 PMC4475444

[30] Taylor MB , Ehrenreich IM . Higher‐order genetic interactions and their contribution to complex traits. Trends Genet. 2015;31:34–40. 25284288 10.1016/j.tig.2014.09.001 PMC4281285

[31] Asomaning K , Miller DP , Liu G , Wain JC , Lynch TJ , Su L , et al. Second hand smoke, age of exposure and lung cancer risk. Lung Cancer. 2008;61:13–20. 18191495 10.1016/j.lungcan.2007.11.013 PMC2515267

[32] Sandoval J , Mendez‐Gonzalez J , Nadal E , Chen G , Carmona FJ , Sayols S , et al. A prognostic DNA methylation signature for stage I non‐small‐cell lung cancer. J Clin Oncol. 2013;31:4140–7. 24081945 10.1200/JCO.2012.48.5516

[33] Bjaanaes MM , Fleischer T , Halvorsen AR , Daunay A , Busato F , Solberg S , et al. Genome‐wide DNA methylation analyses in lung adenocarcinomas: Association with EGFR, KRAS and TP53 mutation status, gene expression and prognosis. Mol Oncol. 2016;10:330–43. 26601720 10.1016/j.molonc.2015.10.021 PMC5528958

[34] Karlsson A , Jonsson M , Lauss M , Brunnstrom H , Jonsson P , Borg A , et al. Genome‐wide DNA methylation analysis of lung carcinoma reveals one neuroendocrine and four adenocarcinoma epitypes associated with patient outcome. Clin Cancer Res. 2014;20:6127–40. 25278450 10.1158/1078-0432.CCR-14-1087

[35] Chen YA , Lemire M , Choufani S , Butcher DT , Grafodatskaya D , Zanke BW , et al. Discovery of cross‐reactive probes and polymorphic CpGs in the Illumina Infinium HumanMethylation450 microarray. Epigenetics. 2013;8:203–9. 23314698 10.4161/epi.23470 PMC3592906

[36] Marabita F , Almgren M , Lindholm ME , Ruhrmann S , Fagerstrom‐Billai F , Jagodic M , et al. An evaluation of analysis pipelines for DNA methylation profiling using the Illumina HumanMethylation450 BeadChip platform. Epigenetics. 2013;8:333–46. 23422812 10.4161/epi.24008 PMC3669124

[37] Benjamini Y , Hochberg Y . Controlling the false discovery rate: a practical and powerful approach to multiple testing. J Royal Stat Soc. 1995;57:289–300.

[38] Ashburner M , Ball CA , Blake JA , Botstein D , Butler H , Cherry JM , et al. Gene ontology: tool for the unification of biology. The Gene Ontology Consortium. Nat Genet. 2000;25:25–9. 10802651 10.1038/75556 PMC3037419

[39] Kanehisa M , Furumichi M , Tanabe M , Sato Y , Morishima K . KEGG: new perspectives on genomes, pathways, diseases and drugs. Nucleic Acids Res. 2017;45:D353–61. 27899662 10.1093/nar/gkw1092 PMC5210567

[40] Wang J , Vasaikar S , Shi Z , Greer M , Zhang B . WebGestalt 2017: a more comprehensive, powerful, flexible and interactive gene set enrichment analysis toolkit. Nucleic Acids Res. 2017;45:W130–7. 28472511 10.1093/nar/gkx356 PMC5570149

[41] Yoshihara K , Shahmoradgoli M , Martínez E , Vegesna R , Kim H , Torres‐Garcia W , et al. Inferring tumour purity and stromal and immune cell admixture from expression data. Nat Commun. 2013;4:2612. 24113773 10.1038/ncomms3612 PMC3826632

[42] Newman AM , Liu CL , Green MR , Gentles AJ , Feng W , Xu Y , et al. Robust enumeration of cell subsets from tissue expression profiles. Nat Methods. 2015;12:453–7. 25822800 10.1038/nmeth.3337 PMC4739640

[43] Heagerty PJ , Lumley T , Pepe MS . Time‐dependent ROC curves for censored survival data and a diagnostic marker. Biometrics. 2000;56:337–44. 10877287 10.1111/j.0006-341x.2000.00337.x

[44] Anastassiou D . Computational analysis of the synergy among multiple interacting genes. Mol Syst Biol. 2007;3:83. 17299419 10.1038/msb4100124 PMC1828751

[45] Watkinson J , Liang KC , Wang X , Zheng T , Anastassiou D . Inference of regulatory gene interactions from expression data using three‐way mutual information. Ann N Y Acad Sci. 2009;1158:302–13. 19348651 10.1111/j.1749-6632.2008.03757.x

[46] Ma S , Kosorok MR , Huang J , Dai Y . Incorporating higher‐order representative features improves prediction in network‐based cancer prognosis analysis. BMC Med Genomics. 2011;4:5. 21226928 10.1186/1755-8794-4-5 PMC3037289

[47] Sethi S , Madden BJ , Debiec H , Charlesworth MC , Gross L , Ravindran A , et al. Exostosin 1/exostosin 2‐associated membranous nephropathy. J Am Soc Nephrol. 2019;30:1123–36. 31061139 10.1681/ASN.2018080852 PMC6551791

[48] Yang CS , Lee JS , Rodgers M , Min CK , Lee JY , Kim HJ , et al. Autophagy protein Rubicon mediates phagocytic NADPH oxidase activation in response to microbial infection or TLR stimulation. Cell Host Microbe. 2012;11:264–76. 22423966 10.1016/j.chom.2012.01.018 PMC3616771

[49] Zhong Y , Wang QJ , Li X , Yan Y , Backer JM , Chait BT , et al. Distinct regulation of autophagic activity by Atg14L and Rubicon associated with Beclin 1‐phosphatidylinositol‐3‐kinase complex. Nat Cell Biol. 2009;11:468–76. 19270693 10.1038/ncb1854 PMC2664389

[50] Matsunaga K , Saitoh T , Tabata K , Omori H , Satoh T , Kurotori N , et al. Two Beclin 1‐binding proteins, Atg14L and Rubicon, reciprocally regulate autophagy at different stages. Nat Cell Biol. 2009;11:385–96. 19270696 10.1038/ncb1846

[51] Kim HP , Wang X , Chen ZH , Lee SJ , Huang MH , Wang Y , et al. Autophagic proteins regulate cigarette smoke‐induced apoptosis: protective role of heme oxygenase‐1. Autophagy. 2008;4:887–95. 18769149 10.4161/auto.6767

[52] Pabón MA , Patino E , Bhatia D , Rojas‐Quintero J , Ma KC , Finkelsztein EJ , et al. Beclin‐1 regulates cigarette smoke‐induced kidney injury in a murine model of chronic obstructive pulmonary disease. JCI Insight. 2018;3:18. 10.1172/jci.insight.99592 PMC6237223 30232271

[53] Kang R , Zeh HJ , Lotze MT , Tang D . The Beclin 1 network regulates autophagy and apoptosis. Cell Death Differ. 2011;18:571–80. 21311563 10.1038/cdd.2010.191 PMC3131912

[54] Zhu L , Chen Z , Zang H , Fan S , Gu J , Zhang G , et al. Targeting c‐Myc to overcome acquired resistance of EGFR mutant NSCLC cells to the third generation EGFR tyrosine kinase inhibitor, osimertinib. Cancer Res. 2021;81:4822–34. 34289988 10.1158/0008-5472.CAN-21-0556 PMC8448971

[55] Sun S , Guo W , Wang Z , Wang X , Zhang G , Zhang H , et al. Development and validation of an immune‐related prognostic signature in lung adenocarcinoma. Cancer Med. 2020;9:5960–75. 32592319 10.1002/cam4.3240 PMC7433810

[56] Stankovic B , Bjørhovde HAK , Skarshaug R , Aamodt H , Frafjord A , Müller E , et al. Immune cell composition in human non‐small cell lung cancer. Front Immunol. 2018;9:3101. 30774636 10.3389/fimmu.2018.03101 PMC6367276

[57] Hwang I , Kim JW , Ylaya K , Chung EJ , Kitano H , Perry C , et al. Tumor‐associated macrophage, angiogenesis and lymphangiogenesis markers predict prognosis of non‐small cell lung cancer patients. J Transl Med. 2020;18:443. 33228719 10.1186/s12967-020-02618-z PMC7686699

[58] Guo Z , Song J , Hao J , Zhao H , Du X , Li E , et al. M2 macrophages promote NSCLC metastasis by upregulating CRYAB. Cell Death Dis. 2019;10:377. 31097690 10.1038/s41419-019-1618-x PMC6522541

