# Supplementary material for: Epigenome‐wide three‐way interaction study identifies a complex pattern between TRIM27, KIAA0226, and smoking associated with overall survival of early‐stage NSCLC
Source: Mol Oncol. 2022 Jan 7;16(3):717–31. doi: 10.1002/1878-0261.13167 (PMC8807353; doi:10.1002/1878-0261.13167)

Supplementary files

Supplementary Table S1. Demographic and clinical descriptions of early-stage NSCLC patients with gene expression data in four international study centers.

Characteristic	Harvard (*N* = 26)	Norway (*N* = 93)	Sweden (*N* = 34)	TCGA (*N* = 456)
Age (years), mean ± SD	66.39±9.45	65.94±9.05	71.85±7.24	66.58±9.14
Sex, n (%)				
Female	11 (42.31)	47 (50.54)	21 (61.76)	182 (39.91)
Male	15 (57.69)	46 (49.46)	13 (38.24)	274 (60.09)
Smoking status, n (%)				
Never	2 (7.69)	11 (11.83)	17 (50.00)	0 (0.00)
Former	15 (57.69)	55 (59.14)	11 (32.35)	316 (69.3)
Current	9 (34.62)	27 (29.03)	6 (17.65)	140 (30.7)
TNM stage, n (%)				
I	20 (76.92)	69 (74.19)	32 (94.12)	294 (64.47)
II	6 (23.08)	24 (25.81)	2 (5.88)	162 (35.53)
Histology, n (%)				
LUAD	17 (65.38)	93 (100.00)	26 (76.47)	221 (48.46)
LUSC	9 (34.62)	0 (0.00)	8 (23.53)	235 (51.54)
Chemotherapy, n (%)				
No	26 (100.00)	76 (81.72)	23 (88.46)	147 (76.17)
Yes	0 (0.00)	17 (18.28)	3 (11.54)	46 (23.83)
Unknown	0	0	8	263
Radiotherapy, n (%)				
No	22 (84.62)	92 (98.92)	26 (100.00)	184 (95.34)
Yes	4 (15.38)	1 (1.08)	0 (0.00)	9 (4.66)
Unknown	0	0	8	263
Adjuvant therapy, n (%)				
No	22 (84.62)	75 (80.65)	23 (88.46)	142 (73.58)
Yes	4 (15.38)	18 (19.35)	3 (11.54)	51 (26.42)
Unknown	0	0	8	263
Survival year				
Median (95% CI)	7.28 (5.41-9.27)	5.27 (5.02-5.65)	3.25 (2.08-4.39)	0.58 (0.50-0.70)
Censored rate, %	26.92	67.74	50.00	76.32

NSCLC: non-small cell lung cancer; TGCA: The Cancer Genome Atlas; LUAD: lung adenocarcinoma; LUSC: lung squamous cell carcinoma; 95% CI: 95% confidence interval

Supplementary Table S2. The association results of variables derived from Cox proportional hazards model adjusted for covariates in NSCLC samples.

Variable	HR	95% CI		*P*
cg00060500*KIAA0226*	1.001	0.951	1.053	0.9787
cg17479956*EXT2*	1.007	0.970	1.017	0.5572

Figure S1. Quality control processes for DNA methylation data. Quality control procedures were applied to Harvard, Spain, Norway, Sweden, and The Cancer Genome Atlas (TCGA) samples using the same pipeline.

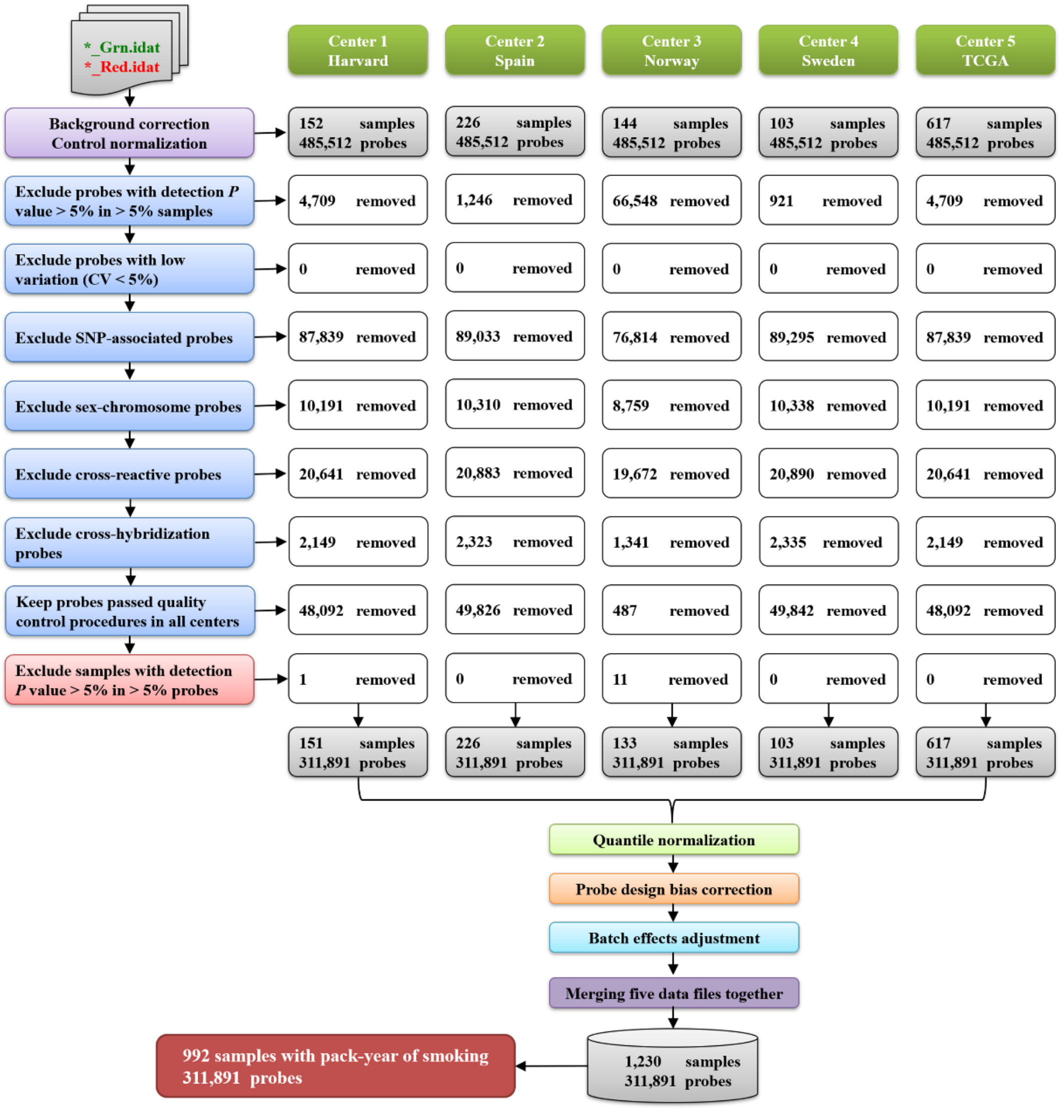

Figure S2. Correlation between DNA methylation probe and its corresponding gene expression. **(A)** *KIAA0226* and cg00060500$_{KIAA0226}$ and **(B)** *EXT2* and cg17479956$_{EXT2}$.

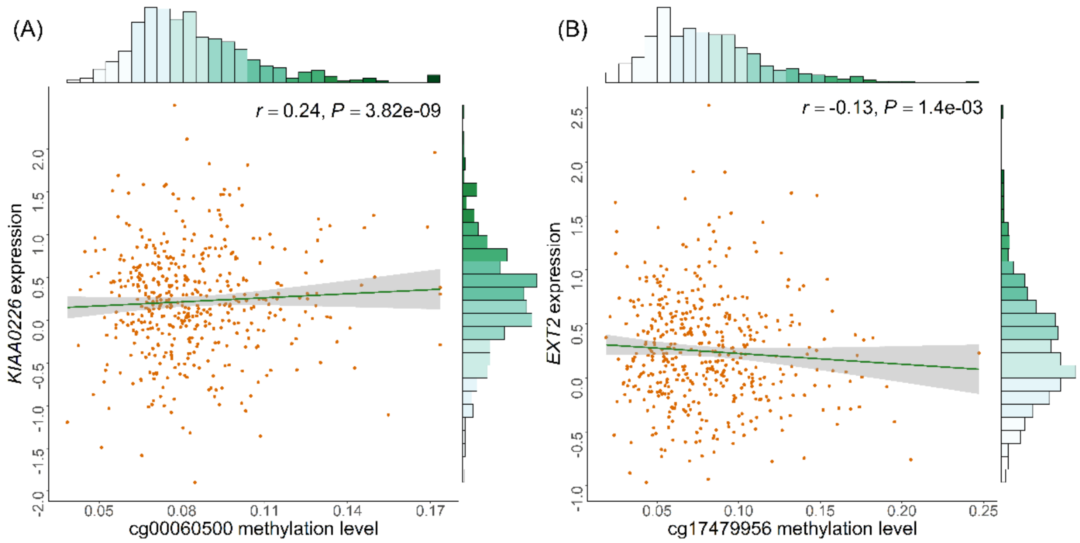

Figure S3. The three-way interaction pattern between pack-year of smoking, cg05293407$_{TRIM27}$ and cg17479956$_{EXT2}$. **(A)** The pattern illustrated by a 3D plot. **(B)** Kaplan-Meier survival analysis of cg17479956$_{EXT2}$ stratified by pack-year of smoking and cg05293407$_{TRIM27}$. From left to right, the number of patients in each subgroup were 266, 211, 282 and 233.

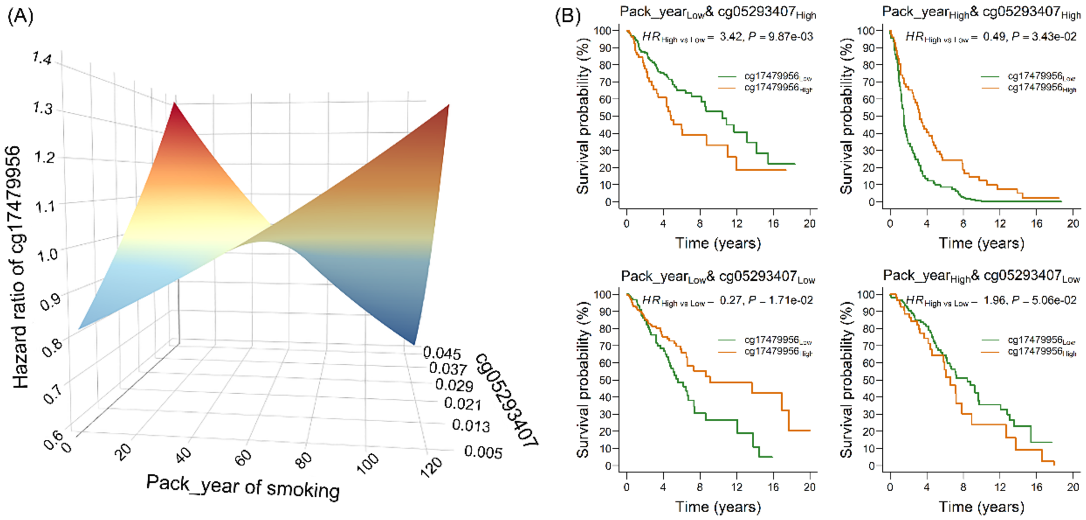

Figure S4. Association between *KIAA0226* and *EXT2*. (A) iTWINS of cg00060500$_{KIAA0226}$ and iTWINS of cg17479956$_{EXT2}$. (B) *KIAA0226* and *EXT2* gene expression. Correlation coefficients were derived from Pearson correlation analysis. Histograms on top and sides were distributions of the two iTWINSs or two gene expressions.

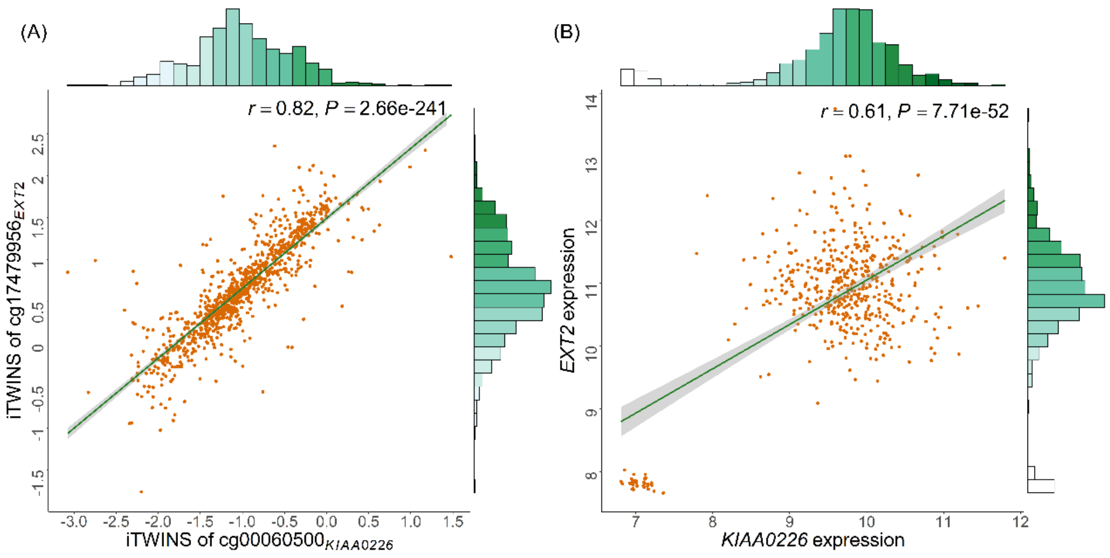

Figure S5. The association analysis between immune score and iTWINS. **(A)** Tested by Pearson correlation analysis, and **(B)** Tested by Kruskal-Wallis rank-sum test.

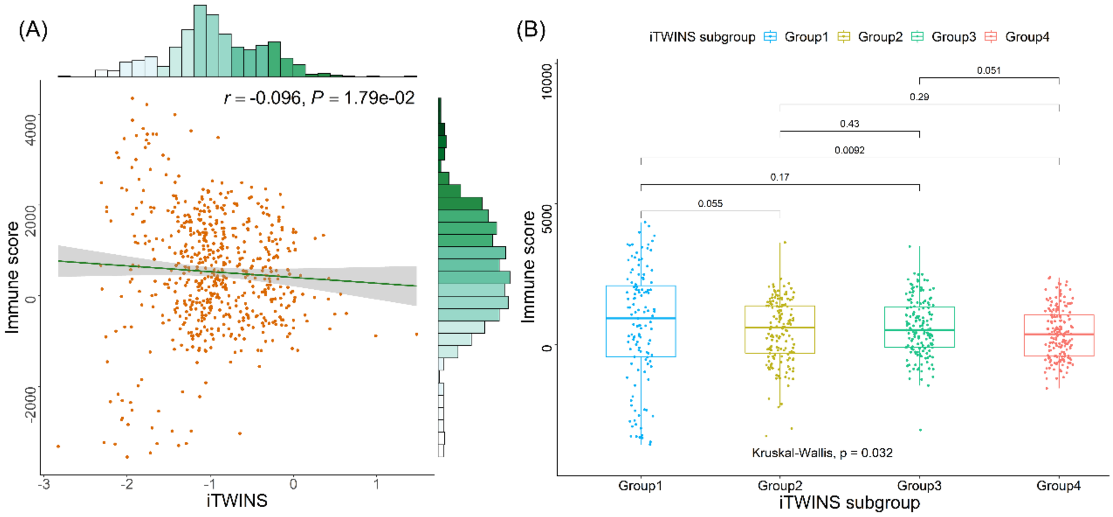

Supplement: Supplementary file 1 — Table S1. Demographic and clinical descriptions of early‐stage NSCLC patients with gene expression data in four international study centers. Table S2. The association results of variables derived from Cox proportional hazards model adjusted for covariates in NSCLC samples. Fig. S1. Quality control processes for DNA methylation data. Fig. S2. Correlation between DNA methylation probe and its corresponding gene expression. Fig. S3. The three‐way interaction pattern between pack‐year of smoking, cg05293407 TRIM27 and cg17479956 EXT2 . Fig. S4. Association between KIAA0226 and EXT2. Fig. S5. The association analysis between immune score and iTWINS. [file MOL2-16-717-s001.pdf]
